# Enhancing Existential Thinking through Death Education: A Qualitative Study among High School Students

**DOI:** 10.3390/bs10070113

**Published:** 2020-07-07

**Authors:** Ines Testoni, Lorenza Palazzo, Ciro De Vincenzo, Michael Alexander Wieser

**Affiliations:** 1 Department of Philosophy, Sociology, Pedagogy and Applied Psychology (FISPPA), University of Padova, 35122 Padova, Italy; ines.testoni@unipd.it (I.T.); lorenza.palazzo92@gmail.com (L.P.); ciro.devincenzo@phd.unipd.it (C.D.V.); 2 Department of Psychology, University of Klagenfurt, Universitätsstr, 65-67, 9020 Klagenfurt am Wörthersee, Austria

**Keywords:** death education, adolescents, hospice, fear of death, terror management theory

## Abstract

The censorship of death-related issues is widespread in contemporary Western culture because the boundary between death and life is substantially managed in medical areas. In the context of Italian educational initiatives, to remove this limitation, 215 high school students in Southern Italy were educated on death through conventional and informal lessons. The students answered a questionnaire with open questions to survey their emotional and reflective experiences. Their answers were qualitatively, thematically analysed to explore how the representation of death can follow a death education course, and if this experience can be managed without harmful effects. The students’ answers narrated how the course reduced their anxiety linked to these themes, on the one hand improving communication between peers by making it more authentic and empathic and, on the other, providing alternative perspectives on life. Indeed, the project offered an opportunity to discuss something strongly heartfelt but rarely faced, and the survey confirmed that the research objectives were fully achieved.

## 1. Introduction

As Gorer [1] first affirmed, death replaced sex as the “forbidden” topic in the second half of the twentieth century. The censorship of death-related issues is widespread in contemporary Western culture; for some authors (e.g., Ariès [2]), this depends on the secularisation and technologization of the boundaries between life and death. Thus, death is completely managed in medical language, and everyday life has lost its ritual practices that used to intertwine community and family relationships in loss and mourning. Now, death and dying are prohibited scenes externalised from private life. Whereas the meaning of life was historically practised informally in families, nowadays it is necessary to plan specific educational activities to recover these conversations [3,4]. However, censorship cannot mitigate the fear of death, instead simply segregating it at an unconscious level without any useful reflection. Indeed, secularised society has reduced faith in an afterlife, which has been one of the most important remedies to death anxiety. As the terror management theory (TMT) demonstrates [5,6,7,8], all cultures develop beliefs that offer a sense that life is meaningful, aetiologies of the universe, prescriptions for appropriate behaviour, assurance of symbolic or literal immortality and death-anxiety management through continuous sense-making in everyday life [9,10]. TMT has obtained both severe criticisms and approvals; however, Martin and van den Bos [11] made perhaps the gravest critique that the theory is not scientific because it is not falsifiable, ignores alternative interpretations and cultural differences and tolerates too broad a range of explanations. Other perspectives propose alternative explanations, including death being threatening because of the uncertainty it entails (e.g., Hohman and Hogg [12]) or because it is uncontrollable [13]. However, TMT researchers have refuted most rebuttals to the theory (e.g., Pyszczynski, Solomon and Greenberg [14]). Although the discussion on the theory is typically heated, some of its claims are certainly valid. Some meta-analyses have confirmed the consistency of the TMT mortality salience hypothesis [15,16,17], especially regarding the relationship between death anxiety and cultural buffers [18,19]. Perhaps most importantly, it is impossible not to consider this theory when confronting death and dying. Using TMT, which highlights the defensive value of cultural perspectives that allow considering death a passage, as some religions do, some death education has already been successfully implemented. Death-related anxiety can be reduced by presenting spiritual and religious content that stimulates thinking about life beyond death [20,21]. Moreover, studies in the psychology of religion and health largely confirm the idea that spirituality and religiosity assist greatly in managing to live with serious illness and death [22,23,24,25,26,27,28].

Therefore, death education may be a path of awareness to encourage reflection on the themes of death and the afterlife. One of its basic ideas is that it is possible to deal with this topic critically and constructively, adapting it to the developmental phase of the individuals to whom it is addressed [20,21,29,30]. It helps young people understand that the media often portray such issues in sensationalist frameworks, focusing on exceptional, cruel, and aggressive factors, while death and dying are real-life experiences that require important abilities to cope with their effects [3,31,32,33,34]. Unfortunately, parents and teachers often find themselves unable to explain these life-and-death messages [35,36]. Adams [37] listed the most common ideas adults have about children and teens’ representations of death: they feel grief less intensely than adults do; it does not make sense to include them in the mourning rites; it is good to hide death events from their eyes; and they do not need to reflect on death because it is the age of light-heartedness. These attitudes nurture misrepresentations of death, which can compromise the ways children and teens treat life and health. The removal of the dialectical relationship between the fear of death and the search for a solution through spirituality and religion can lead to an unconscious suppression of undesirable emotions, and this censorship could diminish the ability to cope with existential anxiety, developing “alexithymia” [38,39], the tendency toward impoverished emotions. The “black hole hypothesis” suggests alexithymia could be related to systematic cultural inhibition on the dialogue about death and dying [36]. For instance, young people are more vulnerable to lacking reflection on mortality to manage its salience [40]. In a broad sense, death education experiences meet this need by promoting reflection on existential themes and exploring contemporary concerns about death and beliefs about the afterlife [3,41,42,43].

The present study qualitatively explores adolescents’ experiences of a death-education course by describing the effects in the first person. The study deeply describes the perceived emotional effects of the course and the possible roles of spiritual/religious accounts and considers whether and how the death-education experience allowed the participants to rethink the value of their lives.

## 2. Materials and Methods

### 2.1. Procedures

The research is based on the results of a death-education project in the southern Italian countryside [21]. It has the general vision of creating a debate to explore different ways of conceptualising death, thus promoting a greater clarification of participants’ existential values. The death-education project and the study started after a proposal shared between teachers, psychologists and students. After group discussion, the students decided to participate and pay attention to their experience in the first person.

The project involved various figures: three specialised psychologists in death education, a psychologist working in a nearby hospice, and an expert in religious education. The death education was divided into “formal work”, which included lessons on death, meditation (Western and Eastern traditions) and religious messages; and “informal work”, which included watching a film on meditation before dying and then creating a movie to elaborate on the sense of the experience. During the informal activities, the students were asked to (a) search for documents about death, loss, spirituality, religiosity, meditation and reflections on the afterlife; (b) to share their feelings and sensations in small-group discussions; (c) to outline and then produce a scenic design to communicate their feelings; and (d) to plan a final presentation for pre-scheduled conferences. Finally, after the classroom activities, the students had a two-hour visit at the local hospice during which they observed the space and could talk with members of the palliative care team. The project lasted five months, and the activities were based on a two-hour weekly plan.

The study followed the American Psychological Association’s Ethical Principles and Code of Conduct, the principles of the 1964 Declaration of Helsinki and its later amendments or comparable ethical standards, and was approved by the Ethics Committee of the University of Padua, Italy (No. 8B6C35ED41F82A9E6BEA6C9094DEE972). The participants were informed about the study’s aims and procedures. The confidentiality of their responses was guaranteed. Informed consent was obtained from all the participants and their parents.

### 2.2. Participants

The project involved 215 students from three different high schools. Among them, 116 were females and 99 males (F = 54 %; M = 46 %) and were between 16 and 20 years old (μ = 17.08; σ = 0.58). Specifically, only one student was 20. Most of the participants were 17 years old (*n* = 164), 20 were 16 years old, 25 were 18 years old and 5 were 19 years old.

Most of the students stated that they were Catholic and believed in God (Yes = 82.8%; No = 17.2%), although the number of people who reported actively participating in religious practices was lower (Yes = 26.5%; No = 73.5%) (Table 1).

### 2.3. Measures

Given that the study was primarily explorative and focused on multidimensional personal (cognitive and emotional) accounts of the participants, a psychological qualitative perspective [44,45] based on the thematic analysis approach was adopted because it offers an accessible, theoretically flexible analysis of narrative data. Thematic analysis is a method for identifying, analysing and reporting patterns (themes) in data. It allows sources to be examined in terms of their principal concepts or themes [46] by integrating two interpretative modalities: top-down (theory-driven) and bottom-up (grounded) [47]. This strategy, which is particularly appropriate in healthcare research [48], implies both predefined themes and themes that emerge as unexpected topics that became clear as the analysis proceed. The study followed three main phases. The first was administrating a qualitative questionnaire at the end of the experience. Students answered the following questions in written form in a setting where they could feel at ease whenever they wanted over the course of three days: What did you earn from this experience? Do you feel you can now deal with the theme of death differently? If so, why? If not, why not? Consistently with the qualitative analysis procedure, we defined a data set which consisted, in this case, of the data corpus to be analysed. Of the 215 participants, only five did not answer the questions, so a total of 210 answers were analysed. The second phase was the data analysis, which used the framework method of thematic analysis. The correspondence between the students’ answers and the aims of the study was carefully studied to identify the central themes and any sub-themes. The third phase had four main steps: coding data; testing emerging understanding; searching for alternative explanations; and writing the report. Personal comments were used to make the experience of each subject clearer so that the interpretation of the material was as detailed as possible. The final interpretative part required total immersion in the texts, analysing all possible connections between the themes, sub-themes and participant groups to underline how everyone gave meaning to their experience. Thematic analysis was performed with ATLAS.ti software, which allowed us to identify thematic networks, examine relevant text segments and link them to particular topics by creating “codes”, “super-codes”, “code families” and “super families” [49,50]. This computer program was optimal for the thematic analysis because it allowed every phase of the analysis to be performed in an elastic, organised way, from coding to recognising the themes and relating the codes. The relationships between the codes were not statistical but semantic, so the links have three types: implicative, causal and opposite. The data were analysed by two independent researchers at the end of each thematic analysis phase to evaluate the convergence of the analysis.

## 3. Results

From the analysis, four thematic areas emerged: “positive change”, “changes between emotion and cognition”, “reflecting on death, life and afterlife” and “openness and confrontation with others”.

### 3.1. Positive Change

This area consisted of students’ perceptions of their cognitive and emotional changes, which were later split into two thematic areas because they involved many elements. In this section, the students reported to deeper understand the meaning of death and the possibility of seeing some good in the experience. For example, Sara said: “It gave me the possibility to reflect on themes related to death and the shortness of life with more consciousness and less sorrow. Of course, the fear of death remains, but now I can try to look at the positive side that post-mortem could reserve us.” In parallel, they described improving their capability to embrace finitude through negotiation with confusion around this theme. The students appreciated the experience at the hospice because they saw how much people care for others and the sense of humanity and affection you can feel. They reported awareness of the importance of doing something that transcends simple medical care and how much the inner reality is as relevant as the physical one. According to some participants, this was a spiritual quality that did not require religion to be defined as such. Some students claimed that, although they were still a little confused and uncertain about most things in their lives and even more about death, they were not so scared anymore but, instead, felt happy about these emotions because they made them feel alive. Both these enhancements were supported by a broad appreciation of life and the will to have new, constructive experiences. Some students said that this experience opened their eyes and made them reflect on the true sense of life, which is sometimes completely forgotten in today’s society—to appreciate life for what it is, to value it and to accept all its negative aspects. For example, Andrea said: “This course gave me the ability to understand how important it is to live every moment to the best and maximum of my possibilities. Additionally, it helped me to consider death not only as a bad thing but also as a possibility for inner growth.” However, increased acceptance of the negative aspects of life also appeared. Some participants stated that the experience was personal growth and understood how important it was to accept every part of life, pleasant or unpleasant, and to seize every moment. Others said that, thanks to this course, they managed to talk with other people about their emotions as they only used to do with their dearest ones. Moreover, they said that they understood that there was no need to fear painful moments in life because, especially with the help of friends, you can always find the strength to face them.

### 3.2. Changes between Emotion and Cognition

**Figure 1:**
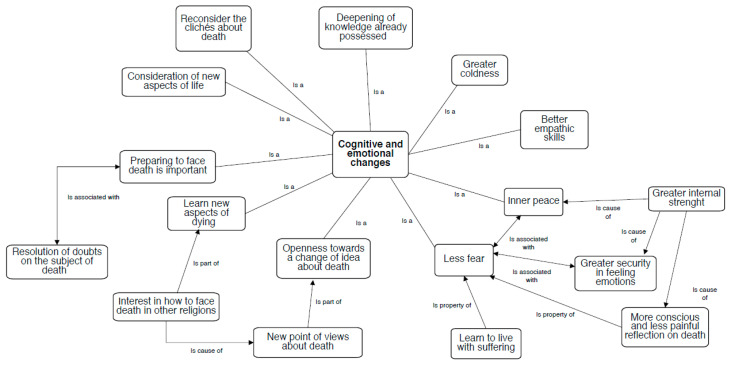
Cognitive and emotional changes.

In this area, both emotional and cognitive changes emerged from the students’ narrative accounts (Figure 1). In particular, a more profound serenity and increased empathic sensation toward those who die were described. Some participants said that this experience gave them different points of view from which to look at death and made them realise that everyone lives it in their own way. Some students also said that they felt they improved their empathic skills because this experience allowed them to realise that others are suffering and dying, which is certainly painful for them and it could be painful for the students in the future, so it is important to be close to those who die and not abandon anyone, especially when there is no hope. About the experience at the hospice, they referred to feeling more serene knowing that there are people who would take care of them in the final phases of their lives and that they could live well in that last phase. This was reinforced by an intense feeling of strength and inner peace by accepting all the emotions, even the negative ones, without censoring them. For example, Marta said: “Even though death scares me terribly, thanks to the support offered by the project, I learned not only to open my horizons but also to accept and try to understand the vision of others regarding death. Understanding that others think of death and explain why we live and die differently from the way I do opened my mind and gave me the desire to think and reflect on these issues. I, therefore, think it is a useful project to be advised to learn how to face life better.” The students referred to feeling more aware that life often contains negative events and situations that cannot be avoided and, although death is difficult to accept, they feel more prepared to face it and even accept their fear of it. Concerning their perception of death, some said that speaking of death was easier; they could approach it from different perspectives more optimistically and serenely, and the thought of death did not frighten them anymore. However, some critical thoughts emerged about themselves, such as realising for the first time that nothing can be taken for granted and that so-called “knowledge” is often uncertain. Knowing and understanding the various ways that humans have managed the awareness of being mortal through religion made the students realise that, since death is an obligatory stop for every person, it is necessary for everyone to prepare during life to face it. Some students referred to this experience being formative because it focused on topics that people often do not dare confront. According to them, it was useful to have an interview with experienced people and with their peers because it enabled them to understand what they really think about the topic, something so absent from many discussions. Moreover, dealing with the theme of death helped them understand not to make partial judgements about life and other people but always to see the beauty and to avoid the error of spending time without emotions.

### 3.3. Reflecting on Death, Life and the Afterlife

The third thematic area is the different representations of death. The first was of death as a passage. Some students had this representation of death before the course, but they reported being able to outline the contours of that passage better after the course. They also said that the course allowed them to believe and hope that after death there would be another life. For many of them, reflecting on death made them feel ready to face the fear of death more serenely than before because they learned that death is not just pain, tears and suffering but also a kind of liberation. Some said they could face death differently because they saw it more as a biological event that cannot permanently disrupt their being because their memory will always return to the hearts of their dear ones.

Although the project was meant to facilitate debate on nihilism, some students maintained their idea that death meant total annihilation. Some claimed to have always considered death the worst possible outcome and that they would continue to think so. For them, dying or losing a person remained an unpleasant end after which they cannot go back—their time is over.

The third dimension of this area pivoted to the existential perspective and concerned the last phase of life. In particular, the importance of paying great attention to those who die was highlighted. For example, Marco said: “Death cannot be avoided, but one can accept and ensure that the patient or he who is about to die does not live this situation badly but that he can enjoy the last days as if they were not really his last. Death is part of life. We must not be afraid of it, so the first step to living a happy life is to consider life exactly as it is, with its joys and sorrows.” The necessity of seeing death as something to be accepted was also underlined. According to some participants, death must not be seen as something negative but rather as something to be accepted, so we must not be upset at the thought of death but continue to live with serenity, making the most of the joys of life. Thanks to this experience, they understood that the passing of one’s own and of loved ones is a part of life, a stage of human existence, and as such we should not fear it but accept it and live with it in tranquillity.

By reflecting on the meaning of existence, issues of quality of life emerged. For example, some students stated that life should be lived to the fullest, day by day, and that every moment is precious; they thought about the importance of spending their time with the people who loved them: their friends, their parents and those who one day they will not have in their lives. Thanks to the experience at the hospice, they realised that sometimes people underestimate life and do not live it fully while others suffer in their last moments. Moreover, this experience gave them the opportunity to reflect upon the value of life. Laura said: “We must grasp everything that happens to us without letting us escape anything, whether beautiful or not, because both teach us something important.” Finally, this experience allowed them to pay attention to other living beings, like Giorgio said: “Little by little I realized I was paying more attention to everything around me. I do not look at a tree with the eyes of before. I think that in every part of what lives there is a heart and a life that flows. I think I have become more sensitive and I try to live every experience better.” Another student, Cecilia, said: “I feel even more at peace with what surrounds me and, therefore, also with death and life. I have always had a positive vision and will continue to have it: with the course, I learned to appreciate even more the beauty of every day that, I am sure, will not be lost and will not be in vain.”

### 3.4. Openness and Confrontation with Others

In this final thematic area, the positive value of diversity emerged from the data. Some students said that the course helped them understand others’ emotions, open themselves to others and reflect on certain situations without feeling alone. This experience allowed them to know their classmates better, discuss deeper issues with them, know their friends’ fears and express their own. They also understood that in the face of life obstacles, people react differently, especially when they are afraid; in the face of death, each of us reacts in a personal, different way. The surprise was discovering that sharing of their darkest thoughts led them closer to each other, less frightened and readier to face pain and speak openly. Carlo said: “This experience has allowed me to touch my feelings, even those of which sometimes one is ashamed or afraid. When we all cried together at the hospice, I understood that it is normal, that I am alive, that crying means digging inside us and touching our feelings. I touched mine and those of my friends, I touched those of others who going to leave and those who still have time to stay here. To talk about life or death for me is to talk about this: about what I am, about what I feel, about what does not make me afraid and about what, like crying, gives me strength. Love for life and for all the people I love feed me.”

## 4. Discussion

This qualitative study addressed the different meanings that a death-education project can promote in high schools, showing that students highlighted no harmful effects. Indeed, their responses evinced great motivation and interest in participating in all the discussions and project activities. Students recognised the relevance and value of an open debate about death-related issues to share concerns and anxieties and to know classmates better. Like what has already been described in the literature [21,34,36,51,52], our results show that students perceived these activities as teaching them to appreciate life, grasp the importance of enhancing one’s own existence and enjoy life fully. They also appreciated the possibility of discussing their greatest fear, namely, the death of loved ones, and sharing those thoughts and feelings with others. Indeed, as already shown [36,53], this is the greatest fear that the thought of death evokes. However, their answers were also consistent with previous studies and perspectives that show that the notion of an afterlife can reduce the anxiety that this thought provokes [21,34,54]. The present study does not report any gender differences related to death anxiety, which, instead, were investigated and discussed in the quantitative part of the project [21]. Moreover, the possibility of communicating one’s own difficult emotions produced both a sense of liberation and greater empathy for others, activating the discussion on everyday meanings, from the simplest to the most important existential themes. Furthermore, they reported the experience, giving them the possibility to clarify their own beliefs regarding the representation of death as a passage or as a definitive end. The main advantage of this study is that it highlights the fact that although death is a thought that causes strong anxiety, as demonstrated by TMT, it is also possible to manage such anxiety by directing emotions and thoughts towards culturally significant existential content. Existential reflection, in fact, if guided by relevant content, can transform fear into a search for meaning [43,55], enhancing the relationships between students that, in this research, were activated by the death-education activities.

Despite all this, the study has some limitations. The first is that the analysed contents were expressed in written and not oral form. This did not allow the researcher to develop a deeper reflection on the issues under consideration. In the future, it will be important to verify with in-depth interviews what death-education experiences might signify. The second limitation is that the present study is not longitudinal and therefore cannot predict possible delayed negative effects of death education. A future study could take advantage of qualitative follow-ups to understand this aspect. The third limitation concerns the sample, since students directly requested the intervention and voluntarily chose to participate.

## 5. Conclusions

This research work stands among the studies that confirm the relevance of death education in high schools, highlighting how adolescents can manage issues on death, their fears and doubts. Their texts highlighted how the course contributed to reducing the anxiety linked to these themes, improved the communication between peers and made it more authentic and empathic, providing different alternative perspectives on life and its sense. Indeed, the project offered the occasion to talk about something that is strongly heartfelt but so rarely faced. Even though someone said the time was not enough, almost all students appreciated the opportunity to have a special setting to express their fears, doubts and ideas. The study showed that coping with death-related issues is a potent means to transmit the interest in scrutinizing their existential concerns and to understand deeply what life meant to them. All this supports what is already shown in the literature, but we want to stress the focus on the interaction between the meanings of death and reflections about the afterlife, which needs much more discussion about religious topics. Indeed, we found that listing several religious perspectives made the students feel more confident about the plurality of modalities that have been used in the various periods and cultures of human history to talk about the same topic, the redeeming subject of religion.

## Figures and Tables

**Table 1:** Participants.

Participants	Age		Gender		Belief in God		Religious Active Participation	
215	**Μ**	**σ**	**F**	**M**	**Yes**	**No**	**Yes**	**No**
	17.08	0.58	116	99	178	37	57	158
